# The cost of type 2 diabetes in Brazil: evaluation of a diabetes care center in the city of São Paulo, Brazil

**DOI:** 10.1186/1758-5996-6-122

**Published:** 2014-11-08

**Authors:** Natalie Botelho Borges, Marcos Bosi Ferraz, Antonio Roberto Chacra

**Affiliations:** Centro de Diabetes da Universidade Federal de São Paulo, Rua Estado de Israel, 639, CEP 04022-001 São Paulo, Brazil

**Keywords:** Type 2 diabetes, Disease cost, Brazil, Outpatient

## Abstract

**Background:**

The worldwide increase of diabetes, a long duration, slow progression disease, impacts health care costs. The aim of this study was to estimate, from the society’s perspective, the annual cost per patient with Type 2 Diabetes (T2DM) at a specialized, outpatient center in the city of São Paulo, capital of São Paulo state, Brazil.

**Methods:**

Data from 209 patients were collected during the years 2009 and 2010 in a São Paulo diabetes care center which is part of the tertiary sector of SUS, Brazil’s National Health Care System. Data were collected by means of interviews and reviews of medical charts, and the quality of life was appraised using the SF36-v2 questionnaire. Direct medical costs were divided in five categories: 1) medication; 2) laboratory tests; 3) hospitalizations and procedures; 4) reactive strips for capillary blood glucose monitoring; and 5) medical consultations. Direct non-medical costs referred to transportation of patient and companion for treatment. Indirect costs included early retirements, sick leave and absenteeism in the workplace. Statistical analysis of the data was performed by the SPSS software, version 17.0.

**Results:**

Our sample comprised 122 women (58%) and 87 men (42%), with mean age of 63 years and average diabetes duration of 13 years. The mean annual cost was US$ 1,844 per patient, out of which US$ 1,012 corresponded to direct costs (55%) and US$ 831 to indirect costs (45%). From the direct medical costs, medications accounted for the greatest proportion (42%), followed by reactive strips (27%), hospitalizations and procedures (14%), laboratory tests and image examinations (7%), as well as medical consultations (4%). Non-medical costs (transportation) corresponded to 7% of the total direct costs. Besides, the results indicated that men have better quality of life than women.

**Conclusion:**

This study demonstrated a high T2DM cost in Brazil, considering the governmental per capita expenses in health care, which accounted for US$ 466 in 2010 (World Health Statistics 2013 96-104 2013). Taking into account the high prevalence of the disease (IDF Diabetes Atlas. 5th edition. 29-48 2012), this survey recommends the enforcement of policies for the prevention of diabetes and its complications, and urges for better allocation of healthcare resources.

## Introduction

The prevalence of diabetes is increasing worldwide [1–3]. The International Diabetes Federation (IDF) estimates that 382 million people lived with diabetes in 2013 [4]. This figure represents a significant percentage of chronic diseases around the globe [5, 6], and according to IDF projections this number will rise to 592 million people in the planet in 2035 [6]. Most of these patients (more than 90%) have Type 2 diabetes (T2DM), a nosological entity that may be prevented [7, 8]. T2DM is characterized by long duration, slow progression and premature mortality from all causes, particularly coronary heart disease [9]. Patients with diabetes use the health care system more frequently and for a longer period than those without this condition [10]. Besides, diabetics have reduced productivity due to the disease or its complications, which impacts not only the National Health Care System, but also the patients’ quality of life [10, 11]. Almost two thirds of that population live in developing countries such as India, China and Brazil, where an increase in the number of diabetic patients in the next two decades [12–15] is foreseen by a number of studies. Among Latin America countries, Brazil has the highest number of people with T2DM. The prevalence of the disease in the adult population from the age of 30 to 69 was estimated in 7.6% by a survey conducted in the end of the 1980’s [16]. More recently, local studies have indicated a 12.1% prevalence in the city of Ribeirão Preto [17] and 13.5% in the city of São Carlos, in the inland of São Paulo state [18], both percentages showing a clear tendency of increase.

Considering the high prevalence of T2DM in Brazil, the number of studies on the cost of this disease is insufficient for an adequate evaluation of the real healthcare needs of that population. Furthermore, this kind of survey constitutes an important tool for better managing the financial resources destined to SUS, which is decentralized and hierarchized – Brazil’s National Health Care System (SUS) is composed of basic health care units, laboratories and hospitals, and aims at providing comprehensive health care to all citizens.

The purpose of this work was to estimate the T2DM annual costs in a specialized diabetes center in the city of São Paulo, Brazil, from the society’s perspective.

## Methods

The study was performed at a university’s outpatient healthcare center which is part of the tertiary level of SUS. For T2DM patients to integrate the study, inclusion criteria were to be over 18 years old and to have regular follow-ups at the outpatient healthcare center for at least one year. The study comprised 209 patients, who were consecutively selected on the basis of consultation schedules from June 2009 to March 2010. All of them were interviewed with the aid of three questionnaires – two of them were filled out by the patient and the other one, by the interviewer. Later, all data were verified by reviewing the patient’s chart.

The first questionnaire was applied to all patients and was divided into three parts. Part one covered demographic, socioeconomic, educational data and productivity in the last 12 months of patients and caregivers; part two referred to clinical data and to the use of healthcare resources along the previous 12 months, such as medical consultations, tests, procedures and hospitalizations, in addition to medications; finally, part three resembled part two, but its data derived from the patient’s chart. Parts two and three were compared, and in case of discrepancies, the data in the patient’s chart prevailed.

The second questionnaire, the Work Limitations Questionnaire (WQL), was applied to all employed patients in the survey [19], in order to assess loss of productivity. The third questionnaire, applied to all participants, was the SF-36v2 Health Survey [20], a generic instrument that has been used all over the world to evaluate quality of life. Already translated to Portuguese and validated in Brazil [21], where this study was conducted, the questionnaire shows several scales, with scores varying from 0 (minimum) to 100 (maximum).

The amount of strips for blood capillary glucose self-measurement (SBMG) was estimated based on the scheme of insulin application. The costs of procedures, hospitalizations, consultations, strips and tests/examinations were calculated based on SUS Unified Table of Procedures (SIGTAP) [22], a comprehensive list of prices practiced by the National Health Care System. As for the price of medications, this study considered the weighted average of the unit value paid by the government at public auctions, as recorded on the price healthcare data bank of the Ministry of Health [23]. Medications considered in our study comprise four groups: antidiabetic, antidislipidemic, antihypertensive and others (diuretic, antiplatelet and drugs for cardiac insufficiency). It was consider the number of tablets taken and the insulin daily dose for the calculation of medication prices.

Indirect costs were calculated by adding the following: a) the results from the WQL questionnaire of the surveyed patients (annual loss rate multiplied by average salary); b) the reported loss of housewives due to diabetes and its complications (estimated as US$ 4.76 per hour multiplied by the additional time required to perform the same tasks); c) the loss due to early retirement, which was assessed considering the last full salary earned when the patient was employed, multiplied by twelve months; and d) loss related to caregivers (workdays lost multiplied by the average family salary). The exchange rate adopted in this study was US$ 1 = R$ 1.837, which was the average exchange rate reported by Brazil’s Central Bank from March 10th, 2009 to April 9th, 2010. The total cost was calculated by adding the direct costs (subdivided into medical and non-medical costs) and the indirect costs.

The statistical analysis was performed using t-Student or ANOVA test for all variables with normal distribution standards. The Wilcoxon or the Kruskal-Wallis test was adopted for the other ones, and the Spearman test was used, with a 5% significance level, in order to verify correlations among the variables.

Patients’ confidentiality was assured. This project was approved by the decision No. 0553/09, taken by the Research Ethics Committee of São Paulo Federal University – UNIFESP.

## Results and discussion

More than half of the sample was composed of women (58%), and the average age of the population was 63 years old. As for schooling, most patients (41.1%) had attended but not finished primary school, while 25.4% had just partially attended or finished middle school, a percentage which reduced to 16.7% in the case of high school and to 12.4% when it came to university. Just 4.3% of the sample had no schooling at all. Regarding labor status, almost half of the population was retired (47%), having average monthly earnings of US$ 576.26 (US$ 19.21 per day). The mean duration of diabetes was 13 years, and 77% of the sample, showed one or more chronic diabetes complications: 34% had retinopathy; 46%, neuropathy; 40%, nephropathy; and 39%, macroangiopathy. Such complications were distributed as follows: 23% of the population did not have any complications from diabetes; 28.2% developed just one; 23% had two; 17.7% had three, and 8.1% had four. The glycated hemoglobin average (HbA1c) at the time of the study reached 8.1%. Since we obtained the exact same value considering the past 5 years and we lacked HbA1c data from some patients at the time of the study, we chose to use the 5-year-long average to increase the number of patients considered. The mean body mass index (BMI) was 29.7 kg/m^2^. Regarding cardiovascular risks, 87% of the sample had arterial hypertension (HAS) and 78% dyslipidemia (DLP). Just 8.6% were regular smokers. These characteristics seem to be related to the T2DM population attending the tertiary level of SUS; similar results were also yielded in another study that characterized the T2DM population of SUS [24]. Demographic data are gathered on Table 1.

**Table 1:** **Demographic and clinical characteristics of the 209 studied patients**

Variable	Male	Female	Total mean (%)	SD
Age (years)	64.35	62.35	63.34	9.98
N (%)	87 (41.6)	122 (58.4)	––	––
**Education**				
Illiterate	2	7	9 (4.3)	0,502
Elementary school	50	36	86 (41.1)	0,455
Middle school	15	38	53 (25.4)	0,496
High school	20	15	35 (16.7)	0,441
University	14	12	26 (12.4)	0,508
**Duration of T2DM (years)**	12.6	13.5	13.19	7.73
**HbA1c (%) of 5 years**	7.81	8.37	8.14	1.60
**BMI (kg/m** ^**2**^ **)**	28.95	30.13	29.65	6.53
**Complications of DM**				
Retinopathy	31	40	71 (34.0)	0.499
Neuropathy	48	49	97 (46.4)	0.502
Nephropathy	35	49	84 (40.2)	0.495
Macroangiopathy	43	39	82 (39.2)	0.502
**Number of complications of DM**				
0	15	33	48 (23.0)	0,468
1	24	35	59 (28.2)	0,495
2	22	26	48 (23.0)	0,504
3	15	22	37 (17.7)	0,498
4	11	6	17 (8.1)	0,493
**Risk factors**				
Arterial hypertension	76	106	182 (87.1)	0.49
Dyslipidemia	66	97	163 (78.0)	0.49
Smoking	7	11	18 (8.61)	0.50

According to the guidelines by the Brazilian Diabetes Association (SBD) [25] and the American Diabetes Association (ADA) [26], a well-controlled diabetic patient must have a HbA1c <7% and, being under this condition, he/she should perform at least 2 HbA1c tests a year. In case of having HbA1c >7% or if medication is changed, it is recommended the same test be performed 4 times along the year. In our study, the average HbA1c was 8.1% (75.5% of the studied population had an average HbA1c >7%), which means that for most of the sample the disease was not well controlled. Furthermore, 18.7% of the population did not get the annual HbA1c test and 37.3% underwent just one test a year (Table 2). This situation may be attributed not only to the patients’ own conduct, but also to the overloaded health care system, which may be sometimes incapable of complying with the demand. Performing tests less frequently than recommended may generate a reduction in the expenses referred to that item, but the lack of control increases the risks of hospitalizations and complications, which have a higher impact on direct costs.

**Table 2:** **Annual utilization of resources by the 209 analyzed patients**

	N*	Mean**	Std. deviation	N*** male (SD)	N*** female (SD)
**Consultations**					
DM Consultations	209	2.23	1.469	205 (2.09)	261 (0.74)
Urology	30	2.17	1.147	45 (0.99)	20(1.40)
Cardiology	67	3.27	3.899	114 (2.50)	110 (4.86)
Dialysis	1	52.00	-----	----	156
Endocrinology	9	3.00	2.000	6 (1.41)	21 (1.89)
Neurology	10	2.20	1.549	9 (1.41)	14 (1.30)
Ophthalmology	31	2.81	2.135	38 (1.32)	49 (2.62)
Nutrition	32	1.41	0.665	21 (0.73)	24 (2.62)
APA	6	3.50	6.124	1	20 (6.00)
Center – DM consultation	37	3.35	2.850	18 (0.43)	61 (2.09)
Podology	4	2.50	1.291	2	8 (1.25)
**Imaging examinations**					
FO	4	0.66	0.568	66 (0.33)	82 (0.29)
Laser therapy	2	0.17	0.660	14 (1.07)	27 (1.16)
ECG	92	1.03	1.362	55 (1.84)	43 (0.66)
Echocardiogram	25	0.96	0.351	13 (0.28)	15 (0.32)
Ultrasound	45	1.04	0.424	19 (0.23)	34 (0.51)
Doppler ultrasound	10	1.10	0.315	4 (0.00)	7 (0.37)
Scintillography	6	0.50	0.548	2 (0.00)	2 (0.49)
Ergometric test	23	0.91	0.288	14 (0.00)	10 (0.37)
**Laboratory exams**	139	1.86	1.666	130 (2.12)	132 (1.13)
Creatine kinase					
Creatinine	177	2.73	5.741	287 (8.26)	200 (1.75)
Glycaemia	174	1.93	1.089	164 (1.35)	193 (0.97)
Postprandial glycaemia	11	0.91	0.302	5 (0.00)	6 (0.35)
HbA1c	174	1.78	0.879	142 (0.95)	175 (0.82)
Total and frac. cholesterol	185	1.88	0.962	156 (0.97)	201 (0.99)
Alanine	139	1.86	1.666	165 (2.48)	145 (0.92)
Aspartate	177	2.73	5.741	165 (2.48)	144 (0.93)
Potassium	174	1.93	1.089	259 (8.82)	180 (1.77)
Sodium	11	0.91	0.302	246 (8.91)	151 (1.84)
Urea	174	1.78	0.879	246 (8.89)	153 (1.70)
Complete hemogram	185	1.88	0.962	200 (9.30)	106 (1.79)
Partial hemogram	22	1.05	0.213	13 (0.00)	13 (0.28)
Uric acid	34	1.24	0.819	28 (0.97)	16 (0.44)
TSH	121	1.53	0.886	79 (0.96)	109 (0.82)
Free T4	51	1.24	0.737	25 (0.98)	39 (0.52)
Gamaglutiltransf.	46	1.96	2.756	62 (3.35)	29 (1.24)
Alkaline phosphatase	30	2.20	3.316	44 (4.07)	22 (1.54)
Total Ca	46	1.76	2.141	46 (2.95)	35 (0.80)
Ionized Ca	18	3.44	7.838	52 (9.10)	10 (1.97)
Microalbuminuria	102	1.25	0.535	51 (0.62)	82 (0.47)
Triglycerides	24	1.04	0.359	10 (0.30)	15 (0.36)
Phosphorus	29	2.17	5.372	44 (7.95)	19 (0.47)

*Number of patients who utilized the resource.

**Number of times that the resource was used per patient, per year.

***Total number of exams or procedures or appointments.

The quality of life assessment (SF-36v2) showed a lower score in the physical (PCS = 45.22) than in the mental domain (MCS = 47.51), being better for men (p-value = 0.0046 in the PCS domain and 0.0034 in the MCS). This had already been observed on a Lithuanian study [27] – as opposed to that survey, however, we found no statistically significant difference in the quality of life due to schooling or family income. Other studies have provided divergent results. The research by Edelman et al. [28], for instance, establishes that diabetes, at its initial stage, does not affect the quality of life. The study by Poljičanin et al. [29] emphasizes, nevertheless, the negative influence of diabetes on the quality of life, pinpointing that patients with diabetes and hypertension show a lower score in both domains of the SF-36 questionnaire, whereas in our study a statistically significant difference was observed only in the physical domain. On the other hand, the study by Venkataraman et al. demonstrates that the quality of life of diabetic patients is mainly affected by a number of complications and not by diabetes itself [30]. In our work, the presence of neuropathy was related to a lower value in the physical domain (PCS), in accordance to the outcome of other studies [30, 31]. A review of the various tools to appraise the quality of life of patients with diabetes confirms that the SF-36 questionnaire is appropriate for this population. However, some researchers recommend the use of specific instruments since they have observed that the SF-36 alone was unable to quantify quality of life modifications despite clear clinical improvements [32]. The results are shown on Table 3.

**Table 3:** **Average values of PCS and MCS (the two main variables in the SF-36v2 questionnaire), according to gender, social class, HbA1c level, hospitalization and complications**

Domain	Variable		Média	Std. deviation	P-value
PCS	Sex	All	45.22	9.82	0.0046
		Female	43.65	10.14	
		Male	47.43	8.95	
	HAS	No	49.41	8.06	0.0 173
		Yes	44.65	9.92	
	DLP	No	47.16	7.79	0.2267
		Yes	44.74	10.28	
	Retinopathy	No	46.03	9.75	0.0900
		Yes	43.71	9.84	
	Nephropathy	No	45.94	9.60	0.2242
		Yes	44.21	10.09	
	Neuropathy	No	46.80	9.33	0.0 160
		Yes	43.43	10.10	
	Macroangiopathy	No	45.61	9.84	0.4232
		Yes	44.45	9.85	
	Hospitalization	No	46.11	9.51	0.0002
		Yes	37.34	9.20	
	HbA1c	1 (4%-6%)	43.90	10.47	0.0345
		2 (6.1%-6.5%)	43.72	12.79	
		3 (6.6%-7.5%)	47.60	9.33	
		4 (7.6%-8.5%)	41.92	9.98	
		5 (>8.5%)	46.03	8.95	
	IBGE* categories	1 (>20 MW)	44.71	12.06	0.9547
		2 (10-20 MW)	46.99	8.45	
		3 (4-10 MW)	45.49	9.33	
		4 (2-4 MW)	45.25	10.25	
		5 (<2 MW)	44.16	9.27	
MCS	Sex	All	47.51	13.31	0.0034
		Female	45.11	14.10	
		Male	50.83	11.42	
	HAS	No	46.53	9.90	0.3 155
		Yes	47.65	13.73	
	DLP	No	47.02	12.44	0.5584
		Yes	47.64	13.61	
	Retinopathy	No	47.16	13.09	0.5614
		Yes	48.18	13.80	
	Nephropathy	No	47.10	12.81	0.3792
		Yes	48.10	14.07	
	Neuropathy	No	48.30	12.69	0.4516
		Yes	46.62	14.00	
	Macroangiopathy	No	47.04	13.10	0.3279
		Yes	48.64	13.22	
	Internment	No	48.41	12.81	0.0 107
		Yes	39.42	15.33	
	HbA1c	1 (4%-6%)	48.44	13.79	0.0964
		2 (6.1%-6.5%)	49.82	11.28	
		3 (6.6%-7.5%)	50.66	12.08	
		4 (7.6%-8.5%)	48.23	13.63	
		5 (>8.5%)	44.31	13.92	
	IBGE categories*	1 (>20 MW)	51.89	8.46	0.3877
		2 (10-20 MW)	50.44	9.94	
		3 (4-10 MW)	49.71	13.06	
		4 (2-4 MW)	46.66	12.15	
		5 (<2 MW)	45.47	14.09	

*Categories defined by the Brazilian Institute of Geography and Statistics (IBGE) according to family income. The average minimum wage (MW) during the period was approximately US$ 263 per month.

The statistically significant variations of the PCS and MCS averages (p-value ≤0,05) are highlighted below.

This study included 22 cases of hospitalizations from 21 patients, with average hospital stays of 9 days. Most hospitalizations (45%) related to macroangiopathy, while others resulted from diabetes or nephric problems, amputations and abscesses. Statistical comparisons between the groups of hospitalized and non-hospitalized patients revealed no significant difference regarding variables such as sex, age, the time it takes to diagnose diabetes, HAS, DLP, HbA1c, school attendance and social class, retinopathy, nephropathy and neuropathy. Macroangiopathy, however, was correlated to a 2.96 higher probability of hospitalization.

The costs of hospitalizations and procedures in this study (US$ 137 per patient/year) amounted to 14% of the direct costs, being slightly lower than those in the Mexican investigation (18%) [33] – the Colombian and the ESCUDI studies did not present this item separately. Previous researches on the cost of illnesses (COI) showed that hospitalizations due to diabetes and its complications are the main item of direct costs – reaching, for instance, 43% in the work *Economic costs of diabetes in the U.S. in 2012* [2] (study based on the prevalence of disease). A similar result was found by the CODE-2 European research [34, 35]. Performed with approximately 7,000 patients in eight European cities, it estimated the annual cost of T2DM at € 2,834 (US$ 3,202) per patient, of which 55% corresponded to hospitalizations and just 7% to antidiabetic medications. However, it is worth noticing that not only the research methodology, but also the health care system and the socioeconomic scenario of the European study are completely different from the Brazilian case, so the results of both works allow for no straightforward comparisons.

Regarding the utilization of resources in our study, the annual average of consultations per patient, according to each specialty, was 3 consultations with a diabetologist, 1 with a cardiologist, 0.3 with a nephrologist, 0.1 with a neurologist, 0.4 with an ophthalmologist and 0.2 with a nutritionist. Detailed results are available on Table 2.

The average annual cost of T2DM was US$ 1,844 per patient, out of which US$ 1,012 (55%) per patient corresponded to direct costs, subdivided into medical costs (US$ 940) and non-medical costs (US$ 72). Expenses with medications (US$ 422 per patient/year) had the highest impact on direct costs (42%). Those medications were divided in four categories: antidiabetics (US$ 113), hypocholesterolinemics (US$ 253), antihypertensives (US$ 25) and others (US$ 31). Among the drugs for glycemic control, NPH insulin was most widely used (87% of the patients), closely followed by metformin (73%). We observed that 42.6% of the patients bought their medication at regular drugstores, either because the health care centers were out of stock, or because drugstores offered more “modern” drugs. However, for the purposes of this study, the costs of medications were calculated as if all the patients had got them from health care centers. In spite of the differences in socioeconomic environment and research methodology, studies performed in Brazil [36], Colombia [37] and Iran [38] also point out medications as the main item in the direct costs of diabetes (Table 4). In the ESCUDI Brazilian study [36], which was the first to investigate the cost of Type 2 diabetes in SUS, medications represented 48% of the direct costs, a result that is similar not only to our findings (42%), but also to those of the Colombian (47%) and of the Iranian (46%) studies, although the percentage of the latter referred only to the patients who had no complications.

**Table 4:** **Results comparison to other T2DM COI studies**

	Cost-of-illness study of T2DM Colombia	Cost-of-illness study of T2DMIran	Rosibel de los Angeles T2DM Mexico	CODE-2 B. Jonsson T2DM Europe	Luciana R. Bahia T2DM Brazil	Natalie B. Borges T2DM Brazil
Year of publication	2009	2011	2010	2002	2011	
Total cost*	2.7 billion	3.78 billion	4.5 billion	EUR 29 billion	––	––
Total annual cost	847	1707	3194**	EUR	2108	1844
per patient*				2834**		
Indirect cost	66%	51%	––	––	33%	45%
Direct cost	34%	49%	––	––	63%	55%
Medication	47%	46%***	––	7%	48%	42%
Laboratory	––	––	––	21%	––	34%****
Procedures and	––	––	18%	55%	––	14%
Hospitalizations Consultations	––	––	––	18%	––	4%

*The costs in all studies are shown in US$, except where indicated.

**Indirect costs were not calculated in these studies.

***This percentage refers only to patients without complications.

****This percentage refers to the cost of laboratory exams (7%) added to those of strips (27%).

In our study, the annual cost of reactive strips for capillary blood glucose monitoring was US$ 269 per patient (27% of the direct costs), higher than hospitalizations and procedures (US$ 137 per patient; 14% of direct cost), when in fact the opposite should be expected. These findings can be explained, however, by the low amount of hospitalizations (22), considering the number of individuals who use reactive strips (123), usually more than once a day. The result also corroborates data of the Brazilian study on T1DM [39]. It shows that health care technology has the greatest impact on the treatment expenses of T1DM, which has a similar treatment to T2DM at its most advanced stage.

The total annual cost of laboratory tests was US$ 71 per patient (7% of the total direct costs) and that of medical consultations was US$ 42 per patient (4%).

In this research, the total annual cost of T2DM was US$ 1,844 per patient, lower than the US$ 2,810 calculated in ESCUDI for patients using SUS’ tertiary sector. Such discrepancy might result from methodological differences, such as the fact that this study did not compute medications for obesity and psychiatric problems, which are responsible for an annual increase in direct costs of approximately US$ 293 per patient in ESCUDI.

The above divergences emphasize the difficulties in comparing the various studies on the cost of the disease and the impossibility to generalize the results, due to differences in aspects such as diabetes demography and epidemiology, clinical practices, distribution and availability of healthcare resources, among other factors [40]. The lack of standardization also hinders comparisons with a large number of studies on the cost of diabetes performed in developed countries, considering that the estimates significantly vary depending upon the methodological approach, the variables taken into account and the health care system in each country [38].

Non-medical expenses (transportation) amounted to US$ 72 per patient per year (7% of the total direct cost), corresponding to an annual average of 11 rides to and from the health care center per patient. We observed that half of the sampled individuals (52%) live in the southern district of the city, where the treatment unit is located, 17% dwell in the eastern district, 5% in the western, 4% in the northern, 1% reside downtown and 11% did not give any address information. The rest of the patients (8%) live in other cities, hence they have higher transportation expenses for each visit. The productivity loss of the workers (n = 31) was 3%, corresponding to US$ 109,460.48 per year. The unemployed patients due to diabetes or its complications and those who reported early retirement and absenteeism in the workplace caused by diabetes and its complications (n = 30) had an annual loss of US$ 48,649.10, while the loss of housewives (n = 14) amounted to US$ 12,162.28 and that of the caregivers (n = 3) to US$ 3,474.94. By adding these numbers we obtained an annual indirect cost of US$ 173,746.80, which gives an average individual cost of US$ 831 per patient per year, representing 45% of the total annual cost. These data are shown on Table 5.

**Table 5:** **Detailed costs of the study**

	Annual costs per patient (US$)	Total annual costs (US$)	% of the direct costs	% of the total costs
**Total cost**	**1,844**	**385,300**	**––**	**100**
**Indirect costs (subtotal)**	**831**	**173,747**	**––**	**45**
**Direct costs (subtotal)**	**1012**	**211,553**	**100**	**55**
**Medical costs** *				
Medical appointments	42	8,688 + 202	4	
Exams	71			
Strips	269			
Hospitalization and procedures (all patients)	137	28,615 + 1,991	14	
Medications**	422	88,182 + 472	42	
**Non-medical costs**				
Transport	72	14,949 + 152	7	

*Costs were calculated using the bottom-up approach with the SUS price as a reference.

**Costs were calculated based on [20] Banco de Preços em Saúde *[Data Bank on Healthcare Prices]*.

This research has some limitations, such as being carried out just at one diabetes care center. Although the facility is located at an important capital city (São Paulo), the results might not reflect the situation throughout the country; possible distortions in our results are nonetheless hard to assess, given the lack of information on key aspects such as regional distribution of patients, access to health care centers and severity of the disease. Since the selection of patients was not ideal, some bias may be noticed, including the predominance of retired people in the sample and the low number of patients having dialysis. The direct costs might also be underestimated since the government has been keeping the prices of the National Health Care System frozen for many years. Hence, the results from this research might not reflect the actual average individual annual cost of T2DM patients at outpatient health care centers all over the country.

In addition to that, the indirect costs might have been underestimated, for some damages caused by diabetes were not considered: there are intangible costs that cannot be measured, such as pain, suffering and an overall decrease in levels of quality of life. According to this study, patients with diabetic neuropathy and arterial hypertension have a statistically significant reduction of quality of life in the physical aspect, while hospitalized patients and women showed lower levels in both domains.

The few existing works on the cost of diabetes in Brazil provide a valuable overview of the matter. However, more studies are needed for better understanding the healthcare needs of this growing population and adequately distributing healthcare resources, which represented 9% of the Gross Domestic Product (GDP) in 2010, and the cost of T2DM found in this study, correspond to 15.7% of the Per capita GDP of 2012, according to the World Health Organization (WHO) [41]. The percentage of the healthcare resources is comparable with that of developed countries such as Australia (9.0%), Finland (9.0), Ireland (9.2), Japan (9.2), Norway (9.3%), Sweden (9.6%) and the United Kingdom (9.6%). Nevertheless, in Brazil, most resources come from private institutions, which provide treatment to a significantly reduced number of patients when compared to the National Health Care System (SUS). In 2010, the government disbursed only 47% of the total amount allocated in the health care sector, while in the aforementioned countries that percentage was significantly higher: 68.5% in Australia, 74.5% in Finland, 69.2% in Ireland, 80.3% in Japan, 85.5% in Norway, 81.0% in Sweden and 83.2% in the United Kingdom.

The relationship between the GDP and the population of each country, i. e., the gross domestic product per capita, must also be taken into account for that analysis. While in Brazil the per capita annual government expenditure on health at average exchange rate was only US$ 466, it reached US$ 3,545 in Australia, US$ 2,947 in Finland, US$ 2,933 in Ireland, US$ 3,179 in Japan, US$ 6,875 in Norway, US$ 3,816 in Sweden and US$ 2,908 in the United Kingdom.

## Conclusion

The growing incidence and prevalence of diabetes confirmed by epidemiologic studies all over the world (IDF) [36] increases the number of people using the health care system of each country. Most of those systems – including SUS in Brazil – are unable to cope with such increase in demand, hence it is essential to enforce policies for the prevention of diabetes and its complications and to better allocate healthcare resources.

## Abbreviations

ADA: American Diabetes Association
BMI: Body mass index
DLP: Dyslipidemia
GDP: Gross domestic product
HAS: Systemic arterial hypertension
HbA1c: Hemoglobin glicosylated
IBGE: Instituto Brasileiro de Geografia e Estatística [The Brazilian Institute of Geography and Statistics]
IDF: International Diabetes Federation
MCS: Mental components of the SF36v2
PCS: Physical components of the SF36v2
SBMG: Self-monitoring of blood glucose
SF36v2: Quality of life questionnaire
SUS: Brazil’s National Health Care System
T2DM: Type 2 Diabetes Mellitus
T1DM: Type 1 Diabetes Mellitus
WQL: Work Limitation Questionnaire.
